# A Metabolomic Study on the Intervention of Traditional Chinese Medicine Qushi Huayu Decoction on Rat Model of Fatty Liver Induced by High-Fat Diet

**DOI:** 10.1155/2019/5920485

**Published:** 2019-02-07

**Authors:** Xiao-jun Gou, Shanshan Gao, Liang Chen, Qin Feng, Yi-yang Hu

**Affiliations:** ^1^Central Laboratory, Baoshan District Hospital of Integrated Traditional Chinese and Western Medicine of Shanghai, Shanghai University of Traditional Chinese Medicine, Shanghai 201999, China; ^2^School of Pharmacy, Shaanxi University of Traditional Chinese Medicine, Yangxian, Shaanxi 712046, China; ^3^Nantong Hospital Affiliated to Nanjing University of Chinese Medicine, Nantong, Jiangsu 226001, China; ^4^Institute of Liver Disease, Shuguang Hospital, Shanghai University of Traditional Chinese Medicine, Shanghai 201203, China

## Abstract

Qushi Huayu Decoction (QHD), an important clinically proved herbal formula, has been reported to be effective in treating fatty liver induced by high-fat diet in rats. However, the mechanism of action has not been clarified at the metabolic level. In this study, a urinary metabolomic method based on gas chromatography-mass spectrometry (GC-MS) coupled with pattern recognition analysis was performed in three groups (control, model, and QHD group), to explore the effect of QHD on fatty liver and its mechanism of action. There was obvious separation between the model group and control group, and the QHD group showed a tendency of recovering to the control group in metabolic profiles. Twelve candidate biomarkers were identified and used to explore the possible mechanism. Then, a pathway analysis was performed using MetaboAnalyst 3.0 to illustrate the pathways of therapeutic action of QHD. QHD reversed the urinary metabolite abnormalities (tryptophan, uridine, and phenylalanine, etc.). Fatty liver might be prevented by QHD through regulating the dysfunctions of phenylalanine, tyrosine, and tryptophan biosynthesis, phenylalanine metabolism, and tryptophan metabolism. This work demonstrated that metabolomics might be helpful for understanding the mechanism of action of traditional Chinese medicine for future clinical evaluation.

## 1. Introduction

Nonalcoholic fatty liver disease (NAFLD) refers to the liver parenchymal cytoplasmic steatosis and fat storage as characteristics of the clinical pathological syndrome excluding alcohol and other determinants of liver damage [1]. With the improvement of living standards and lifestyle changes, the prevalence of diseases such as obesity, insulin resistance (IR), hyperlipidemia, diabetes, and metabolic syndrome contributes to the development of NAFLD. The increasing incidence of NAFLD has become a global medical and public health problem [2–4]. NAFLD can be developed from the original nonalcoholic fatty liver (NAFL) to nonalcoholic steatohepatitis (NASH), even to the later stage of liver fibrosis, liver cirrhosis, and advanced hepatocellular carcinoma in the absence of effective intervention [5]. At present, NAFLD has become the most common cause of chronic liver disease and hepatic enzyme abnormality in western developed countries such as Europe and the United States. Epidemiology shows that NAFLD involves an average of 10% to 24% of the world's population and the prevalence rate in obese people is up to 57% - 74%. About 50% of NAFLD patients can develop into nonalcoholic steatohepatitis after 4~13 years, and 40% of the patients develop into hepatic fibrosis. The incidence of NAFLD is significantly higher than that of hepatitis B, hepatitis C, and alcoholic liver disease, and it has become one of the most common diseases [6, 7]. In recent years, the prevalence of NAFLD in China and the Asia Pacific region has increased year by year, and the adult incidence in developed areas of China can reach 15% or even higher [8]. The common risk factors of NAFLD are hypertriglyceridemia, obesity, and type II diabetes, and the pathophysiological process of NAFLD is extremely complex. Its exact cause and mechanism are not yet clear.

Lifestyle changes, such as weight loss, diet changes, and physical activity, can promote the treatment of NAFLD [9]; however, most people find it difficult to adhere to exercise [10]. In addition, although some pharmaceutical preparations have been approved for NAFLD, many drugs have potential side effects or only show efficacy for individual patients [11]. Therefore, researchers have become increasingly interested in finding natural products to treat NAFLD from diet and natural plants/herbs. Many natural products have been used to treat NAFLD due to their obvious liver protection, hypoglycemia, antihyperlipidemia effect, and negligible side effects [12].

QHD is an effective prescription for NAFLD treatment based on our long-term clinical practice. It is made up of five traditional Chinese medicines, including* Herba Artemisiae Capillaris, Rhizoma Polygoni Cuspidati, Herba Hyperici Japonici, Rhizoma Curcumae Longae, gardenia jasminoides ellis*. It has the functions of clearing heat, removing dampness and detoxifying, promoting blood circulation, and dispersing blood stasis. In nearly a decade, animal experiments have repeatedly confirmed that QHD has a significant preventive effect on fatty liver induced by high-fat diet alone and carbon tetrachloride (CCL_4_) combined with a high-fat and low-protein diet in rats [13, 14]. Clinical studies have also shown that QHD can significantly improve the degree of fatty liver in patients with nonalcoholic steatohepatitis and effectively alleviate the main clinical symptoms of abdominal distention, flank pain, and other symptoms in patients with fatty liver and improve the efficacy of TCM syndromes. QHD has markedly reduced liver damage indicators, such as serum ALT, AST, TG, TC, and LDL-c levels in patients [15]. In the earlier period, our group investigated the mechanism of QHD in some aspects of the pathological mechanism of nonalcoholic fatty liver (NAFL), indicating that the mechanism of QHD in preventing and treating NAFL involves regulation of adenosine monophosphate-activated protein kinase (AMPK) activity, elevation of adiponectin, regulation of intestinal flora, reduction of free fatty acid (FFA) toxicity, etc. [16–19]. Traditional Chinese medicine (TCM) compound has the characteristics of multipathway and multitarget pharmacology, and the mechanism of action is very complex. These results suggested that QHD was a promising compound with good druggability. However, these studies are limited to the exploration of certain specific pathological aspects and elucidation of curative effect and mechanism of treatment of NAFLD is not sufficient for QHD.

Metabolomics aims to establish the metabolic profiles of low molecular weight endogenous metabolites in the biological system through modern analytical techniques [20]. Metabolomics is a branch of systems biology that focuses on the overall metabolite spectrum in various biological samples, such as urine, plasma, or tissues [21]. This research strategy is consistent with the overall and systematic nature of TCM [22]. Due to these advantages, more and more attention has been paid to the mechanism of TCM prescriptions revealed by metabonomics, such as Huangqin Decoction and Liuwei Dihuang Decoction [23, 24]. Some analytical tools have been used in metabolomic analysis, such as nuclear magnetic resonance spectroscopy (NMR), mass spectrometry (MS) and gas chromatography (GC/MS), liquid chromatography (LC/MS), and capillary electrophoresis (CE/MS). Among them, GC/MS provides sufficient separation of complex sample, high sensitivity and metabolite resolution, and easy access to NIST database, which has developed into a popular and useful analysis technology in metabolomic research [25]. Our previous serum and liver tissue metabolomic studies suggest that the effects of Qushi Huayu Decoction on fatty liver may involve regulation of beta-alanine metabolism, alanine, aspartate, and glutamate metabolism, glycine, serine, and threonine metabolism, pyruvate metabolism, and citrate cycle [26]. Owing to the limited time and the economic difficulty, we previously studied only the samples of serum and liver tissue and did not observe a time-dependent dynamic change in the metabolomic profile. Meanwhile, urine, as important biological samples, may be attractive for biomarker investigation to provide a new insight into the progression of fatty liver and the therapeutic basis of QHD. In addition, the urine sample is the end product of the body's metabolism. It is noninvasive and dynamic and can be used to observe changes in metabolic profiles during animal modeling. The results of urine metabolomics are also a complement to the results of serum and liver tissue metabolomic.

In this work, based on previous studies of serum and liver tissue metabolomics, we used a GC-MS-based urine metabolomic method combined with pattern recognition and pathway analysis to analyze their metabolic characteristics of control group, model group, and QHD group. In addition, we also introduced a typical metabolic pathway network to explain biochemical mechanisms, thus providing a multiobjective interpretation of the effect of QHD on NAFLD.

## 2. Materials and Methods

### 2.1. Chemicals

N,O-bis(trimethylsilyl)trifluoroacetamide (BSTFA+1%TMCS) and methoxyamine hydrochloride were obtained from TCI (Shanghai) Chemical Industry Development Co., Ltd. Myristic acid and urease were purchased from Sigma-Aldrich (St. Louis, MO, USA). Heptane, pyridine, and methanol are of analytical grade from Shanghai Aladdin Biochemical Technology Co., Ltd. Commercial kits for the determination of alanine aminotransferase (ALT), aspartate aminotransferase (AST), low density lipoprotein cholesterol (LDL), high density lipoprotein cholesterol (HDL), and triglyceride (TG) came from Nanjing Jiancheng Institute of Biotechnology (Nanjing, China).

### 2.2. Preparation of QHD

QHD was prepared as described in an earlier report [17]. The concentration for the final stock solution of QHD extract was adjusted to 0.93g/crude herb/mL, and the quality control of QHD was shown in the additional material files (available here).

### 2.3. Experimental Animals

The protocol was approved by the Animal Experiment Ethics Committee of Shanghai University of TCM, and the study was carried out under the Guidelines for Animal Experimentation of Shanghai University of TCM (Shanghai, China). Thirty male Sprague-Dawley rats (weighing 170±20g) were commercially obtained from Shanghai Experimental Animal Center of Chinese Academy of Sciences (Shanghai, China), and the food materials for the animals were commercially obtained from Shanghai Laboratory Animal Center (Shanghai, China). All the animals were fed at a temperature ranging from 23°C to 24 23°C and humidity of 60%  ± 10% in a 12/12-hour light-dark cycle. The rats were fed with certified standard chow and tap water ad libitum for 1 week acclimation.

### 2.4. Drug Treatment and Sample Collection

After 1 week of adaptation, 30 rats were randomly divided into the following three groups of 10 each: control group (feeding with normal diet for 8 weeks, and drinking tap water normally for the first 4 weeks, orally administered with saline daily on each of the last 4 weeks), model group [feeding with high-fat diet (10% lard+2% cholesterol + 88% normal diet) for 8 weeks, and drinking tap water normally for the first 4 weeks, orally administered with saline daily on each of the last 4 weeks], and QHD group [feeding with a high-fat diet for 8 weeks, drinking tap water normally for the first 4 weeks, orally administered with tap water ad libitum on each of the first 4 weeks, dosed orally with QHD 0.093gkg^−1^day^−1^ on each of the last 4 weeks according to literature [14] with some modifications].

The samples of overnight (12h) urine from all the rats were collected in metabolism cages at 0 weeks before modeling, 4 weeks before QHD administration, 6 weeks during QHD administration, and 8 weeks after QHD administration throughout the experimental period and were immediately stored at −80°C after centrifugation at 3000rpm for 10min to remove the residues. All the rats were sacrificed by anesthesia with 2% sodium pentobarbital (3mLkg^−1^). The serum samples were collected, centrifuged at 3000rpm for 10min, and stored at −80°C until analysis. The livers were immediately weighed and washed with cold normal saline. The samples from the right liver lobes were then fixed in 10% neutral formalin for histological analysis. The samples from the left liver lobes were immediately frozen at –80°C for subsequent analysis.

### 2.5. Analysis of Liver Function and Pathological Examination

Serum samples were used to measure the levels of triglycerides (TG), low-density lipoprotein cholesterol (LDL-c), high-density lipoprotein cholesterol (HDL-c), alanine aminotransferase (ALT), and aspartate aminotransferase (AST) by using ELISA Kit according to the manufacturer's instructions. The liver TG was also measured by using commercial kits as per the manufacturer's instructions. Liver tissue was fixed with formalin and embedded in paraffin, sectioned, and stained with hematoxylin and eosin (HE) and Oil-Red O, respectively, according to the standard protocol.

### 2.6. Urine Sample Preparation and Analysis

The analysis of urine samples was performed by using a published method [27] with some modifications, and detailed steps were shown in the additional material files.

### 2.7. GC/MS Analysis

Each 1*μ*l aliquot of the analytes was injected into an Agilent 6890N GC/5975B inert MS detector (Agilent Technologies, Santa Clara, CA, USA). The separation was achieved on an HP-5MS capillary column (30m x 250*μ*m internal diameter, 0.25*μ*m film thickness, 5% phenyl methylpolysiloxane bonded and cross-linked; Agilent J&W Scientific, Folsom, CA, USA). The MS parameters used included the following: the injection and interface temperature were set at 260°C, and the ion source was adjusted to 200°C. The GC oven temperature was kept at 70°C for 2min, ramped at 5°Cmin^−1^ to 160°C, and finally held at 240°C at a rate of 10°C min^−1^ for 6 min. Helium was used as the carrier gas at a flow rate of 1ml/min. The electron energy was 70 eV, and detection was carried out in full scan mode (m/z 30-600) with a solvent delay of 5 min.

### 2.8. Data Processing

Unprocessed GC/MS raw files were converted to NetCDF format via DataBridge (PerkinElmer Inc, USA), and then the baseline correction, peak discrimination and alignment, and retention time correction were performed by the XCMS toolbox (https://xcmsonline.scripps.edu/) with default settings. The result table (TSV file) is exported to Microsoft Excel, and all data are standardized to spectral sum before multivariate analysis. Multivariate statistical analysis tools were used to analyze data by pattern recognition. The SIMCA-P11.5 software package (Umetrics AB, Umea, Sweden) was used for principal component analysis (PCA) and partial least squares discriminant analysis (PLS-DA). In addition, an unpaired Student t-test was used to assess significant differences in discriminant scores or concentrations of different metabolites obtained from PLS-DA modeling between the model group and the control group. The concentration of each metabolite was expressed as the ratio of its peak area value to that of the selected internal standard peak area of myristic acid.

### 2.9. Statistics

SPSS 21.0 statistical software package (SPSS, Chicago, USA) was used for quantitative analysis and expressed as mean ± standard deviation. Student-Newman-Kerr one-way analysis of variance was used to analyze the statistical differences among groups. The results of P < 0.05 were considered as statistically significant.

## 3. Result

### 3.1. Body Weight

**Figure 1:**
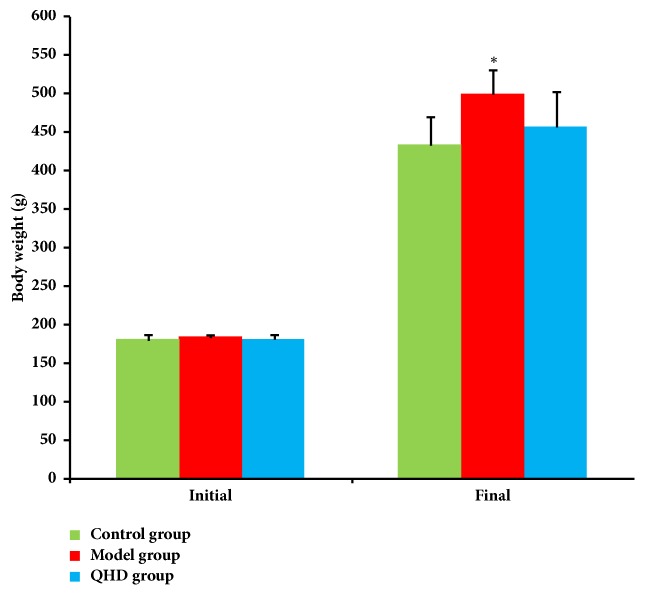
Body weight from animals of different groups, ^*∗*^* p*<0.05, compared with control group.

As shown in Figure 1, there was no significant difference in initial body weight between the 3 groups. After 8 weeks, compared with the control group, the weight of the model group increased significantly (*p*< 0.05), while the weight of the QHD group decreased, and there was no significant difference compared with the model group.

### 3.2. TG Level in Liver

**Figure 2:**
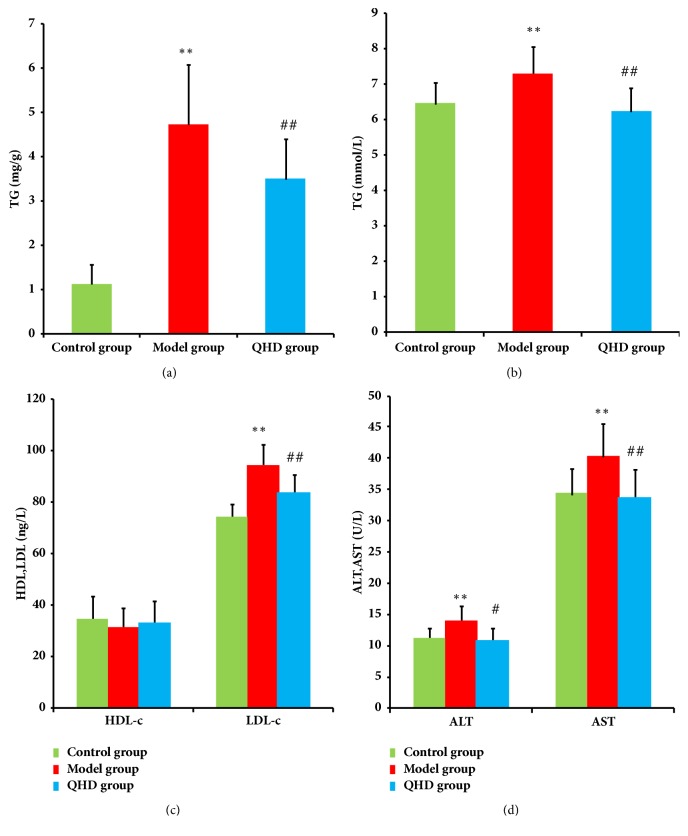
Hepatic levels of TG (a) and serum levels of TG (b), HDL and LDL (c), AST and ALT (d). ^*∗*^*p*<0.05, ^*∗∗*^*p*<0.01 compared with control group, ^#^* p*<0.05, ^##^* p*<0.01 compared with model group.

As shown in Figure 2(a), after 8 weeks on a high-fat diet, the rats in the model group exhibited increased TG levels compared to the rats in the control group (*P *< 0.01). After treatment with QHD, the level of TG was decreased compared to the rats in the model group (*P* < 0.01).

### 3.3. Serum Levels of ALT, AST, TG, LDL, and HDL

At the end of the experiment, the serum levels of ALT, AST, TG, and LDL in the model group were significantly higher than those in the control group (*P *< 0.01). Compared with the model group, QHD significantly reduced ALT, AST, TG, and LDL (*P *<0.05 or* P* <0.01), respectively. There was no significant difference in serum HDL between QHD and model group [Figures 2(b), 2(c), and 2(d)].

### 3.4. Histopathological Changes of the Liver

**Figure 3:**
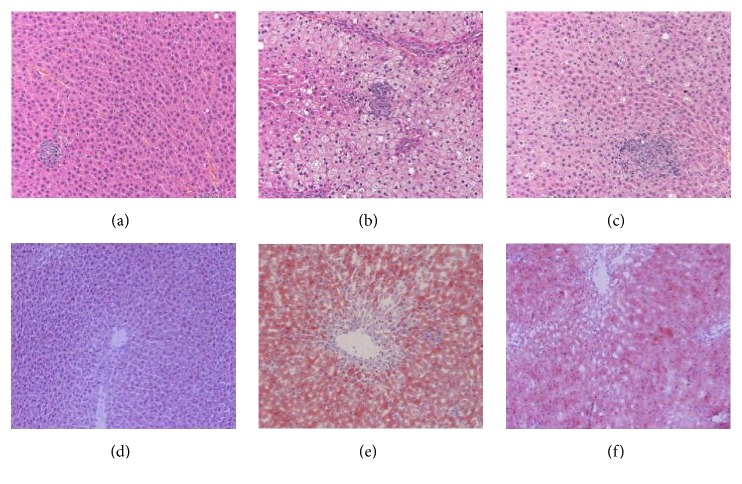
Liver sections showing steatosis and inflammation with H&E staining (×200) and Oil Red staining (×200). H&E staining from (a) control group, (b) model group, and (c) QHD group. Oil Red staining from (d) control group, (e) model group, and (f) QHD group.

Histopathological changes of liver were examined in H&E stained sections. Liver samples of model rats showed* degeneration of hepatocytes, abundant* fat deposition, inflammatory infiltration, prominent hepatocyte balloons, and a single large vacuole in the cytoplasm of hepatocytes. Histopathological features of fatty liver were observed in QHD group, but these symptoms were alleviated. No histopathological signs were found in the control group [Figures 3(a)–3(c)]. Oil red O staining of liver tissue showed that no red lipid droplets were seen in the normal group. Compared with the normal group, liver steatosis was obvious, lipid droplets were large, the central area of the lobule was darkly stained, and the marginal area was lightly stained in the model group. The above changes in the QHD group were obviously reduced [Figures 3(d)–3(f)]. These phenomena indicate that the rat fatty liver model induced by high fat diet was successful and the histopathological status was improved with the use of QHD.

### 3.5. Metabolomic Study

#### 3.5.1. GC/MS Spectra of the Three Groups

**Figure 4:**
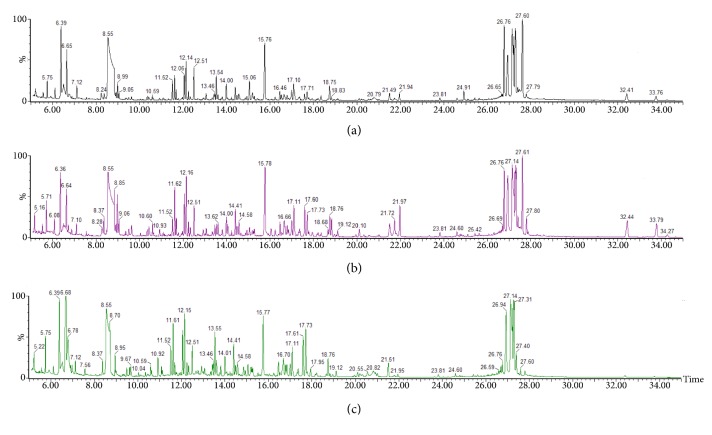
Typical GC/MS spectra of urine samples from (a) control group, (b) model group, and (c) QHD group.

A typical GC/MS total ion current (TIC) chromatogram of the urine samples from the control, model, and QHD groups was illustrated in Figures 4(a), 4(b), and 4(c), respectively. The visual inspection of the spectra revealed some obvious differences, but the complexity of GC/MS spectra prevented further comparison between classes. Thus, XCMS and Microsoft Excel software were used to pretreat the GC/MS spectra and obtained a three-dimensional matrix (RT–m/z pairs), 30 sample names (observations), and peak area percentage (variables). The resulting data set was subsequently analyzed to extract useful information by multivariate statistics including PCA and PLS-DA.

#### 3.5.2. Analysis of Metabolic Profiles and Identification of Significantly Changed Metabolites

**Figure 5:**
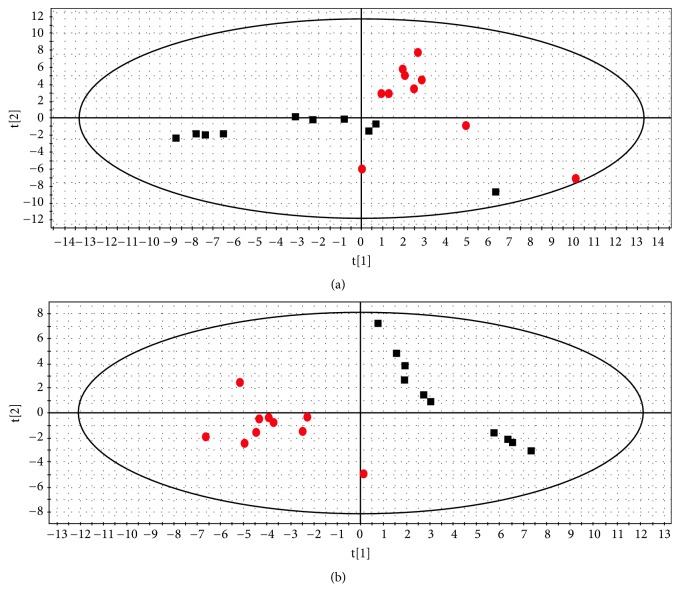
Score plot of control group (black square) and model group (red circle) from a PCA model (a) and a PLS-DA model (b).

In this work, typical GC/MS TIC chromatograms of urine samples from the control, model, and QHD groups are shown in Figures 4(a), 4(b), and 4(c), respectively. Subtle changes can be found using pattern recognition methods such as PCA and PLS-DA. PCA and PLS-DA are two of the most popular pattern recognition methods for obtaining information about the classification and identification of metabolites. PCA is an unsupervised method used as the first step in the separation process to filter out noise and reduce the dimension of the observed data. PLS-DA is a monitoring method similar to PCA in principle for improving classification performance [28]. PCA displays the poor separation between the control group and the model group (Figure 5(a)).

**Figure 6:**
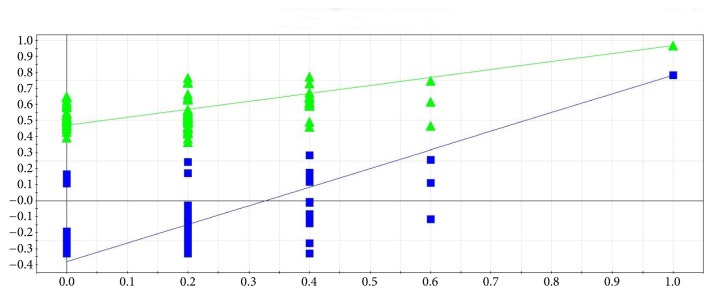
Two hundred permutations were performed, and the resulting R2 and Q2 values were plotted. Green triangle: R2; blue square: Q2. The green line represents the regression line for R2 and the blue line for Q2.

To improve the classification of the model and control groups, a PLS-DA model was performed. As shown in Figure 5(b), the samples in the model and control groups were clearly separated; and the parameters of the modeling such as R^2^X, R^2^Y, and Q^2^Y were 0.61, 0.97, and 0.78, respectively, indicating that the metabolic profile of the rats in the model group was different from those in the control group. A permutation test was conducted to further validate the model [29]. After 200 permutations, the intercept values of R2 and Q2 are 0.48 and -0.38. The negative value of Q2 intercept indicates the robustness of the model, which shows a low risk of overfitting and reliability (Figure 6).

**Figure 7:**
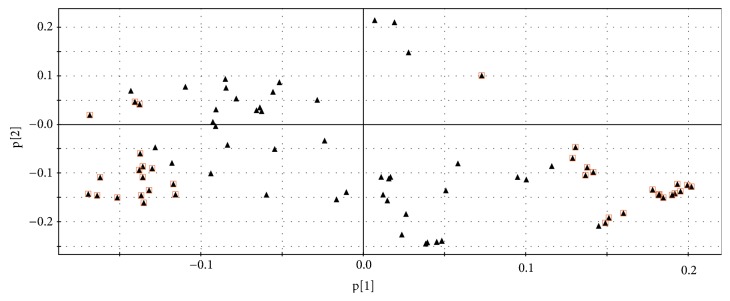
Loading plot from PLS-DA model classifying model obtained from control group and model group. Red square: difference variables.

The PLS-DA loading plot (Figure 7) showed the variables that had contributed strongly to the separation of the groups, the significantly changed metabolites that were the furthest one from the origin in the loading plot. According to VIP (the variable importance in the projection)>1[30], 36 difference variables were found. Next, based on P value of Student t-test (*p*< 0.05), and matching value from the NIST library for more than 800 (out of 1000) endogenous metabolites, 12 significantly changed metabolites could be identified from the loading plot [31]. Among them, 4 were decreased in model rats, whereas the other 8 were upregulated. The results were summarized in Table 1.

#### 3.5.3. The Influence of QHD on the Urinary Metabolic Profiles of High-Fat Diet Rats

**Figure 8:**
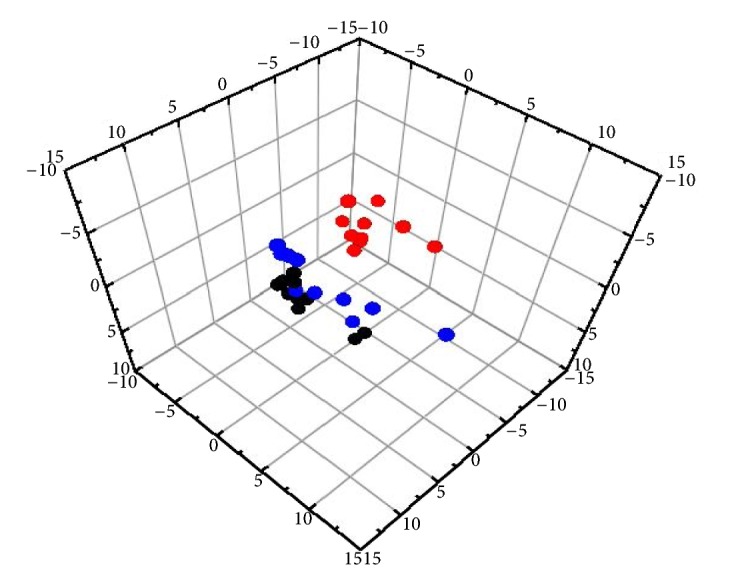
Score plot of 3D-PLS-DA model obtained from control group (black circle), model group (red circle), and QHD group (blue circle).

To evaluate the influence of QHD on the urinary metabolic profiles of the high-fat diet rats, a three-dimensional PLS-DA scores plot was built to depict the general variation between the control and model groups with QHD intervention. The scores plot (Figure 8) showed clear separation of the model and control groups. The results indicated that the urinary metabolic pattern was significantly changed in the high-fat diet treated-model group. Also, the QHD treated-group located between the model and control groups, and it was much closer to the control group, implying that QHD did effectively prevent the progression of fatty liver and regulated the perturbed metabolism.

The mean level of the 12 metabolites showed a tendency to normal at different degrees after taking QHD (Table 1). Among these metabolites, tryptophan, uridine, and phenylalanine in the QHD-treated group were completely reversed to levels in the control group (Table 1). It was revealed that the concentrations of these metabolites, which were altered in the rats of model group, had the tendency to come back from the model group to the control group after taking QHD. Above results confirmed that the disturbed urine metabolites due to high-fat diet were regulated by QHD. The results of liver function tests, histological changes, and these change in urine metabolic pattern confirmed that QHD had obvious anti-liver fatty effect.

#### 3.5.4. Time-Dependent Changes of Metabolic Profile

**Figure 9:**
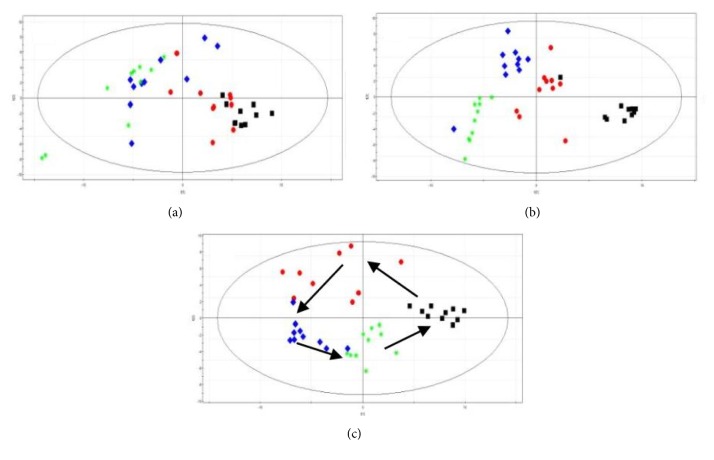
Score plot from PCA derived from the GC/MS profiles of urine samples obtained from (a) control group, (b) model group, and (c) QHD group. The plot shows the trajectories of metabolite patterns at different time points. Black square: 0 weeks before modeling, red circle: 4 weeks before QHD administration, blue diamond: 6 weeks during QHD administration, and green asterisk: 8 weeks after QHD administration.

In this study, time-dependent changes of metabolic profile of urine samples from control group, model group, and QHD group rats were obtained at 0 weeks before modeling, 4 weeks before QHD administration, 6 weeks during QHD administration, and 8 weeks after QHD administration (Figure 9).

In the PCA score plot, there was no significant change in the control group. The metabolic patterns of rats in the model group were significantly different at different time points, and there was a deviation trend from 0 weeks before modeling to 6 weeks after QHD administration, which showed a metabolic change induced by high-fat diet. In the QHD group, the metabolic pattern 4 weeks before QHD administration significantly deviated from the 0 week before modeling. Metabolic patterns at 6 and 8 weeks showed a reversal trend at 0 week before model state with the treatment of QHD. This result suggests that QHD has the potential to correct deviations caused by high-fat diets.

#### 3.5.5. Metabolic Pathway Analysis of QHD on Rat Model of Fatty Liver Induced by High-Fat Diet

**Figure 10:**
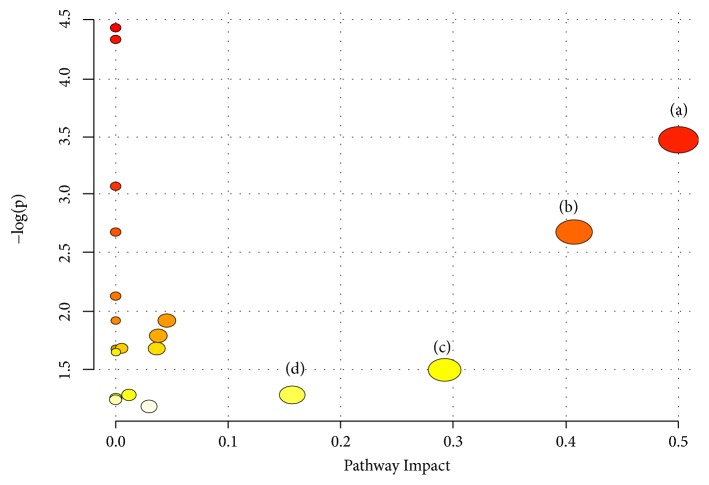
Summary of pathway associated with rat model of fatty liver induced by high-fat diet. (a) Phenylalanine, tyrosine, and tryptophan biosynthesis, (b) phenylalanine metabolism, (c) glycine, serine, and threonine metabolism, and (d) tryptophan metabolism.

In order to identify the most relevant pathway, the threshold of the influence value of the pathway analysis by MetaboAnalyst 3.0 is set to 0.10 [32]. In this study, 12 potential biomarkers associated with group separation were entered into online system MetaboAnalyst. There are four significant pathways associated with rat model of fatty liver induced by high-fat diet. The top 4 metabolic pathways included (a) phenylalanine, tyrosine, and tryptophan biosynthesis, (b) phenylalanine metabolism, (c) glycine, serine, and threonine metabolism, and (d) tryptophan metabolism (Figure 10).

**Figure 11:**
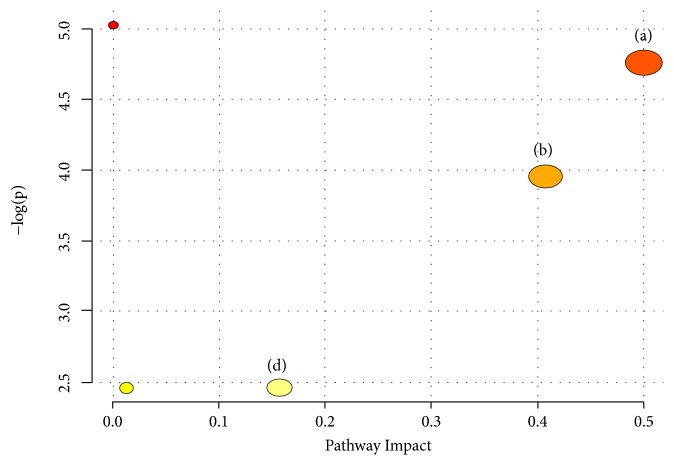
Summary of pathway associated with effect of QHD on rat model of fatty liver induced by high-fat diet. (a) Phenylalanine, tyrosine, and tryptophan biosynthesis, (b) phenylalanine metabolism, and (d) tryptophan metabolism.

In this study, the metabolic changes related to fatty liver on the QHD treatment group were analyzed. Compared to the model group, three different metabolites were completely reversed to levels in the control group (Table 1). According to the MetPA analysis (Figure 11), phenylalanine, tyrosine, and tryptophan biosynthesis, phenylalanine metabolism, and tryptophan metabolism were significantly associated with effect of QHD on rat model of fatty liver induced by high-fat diet (Figure 11).

## 4. Discussion

The present study describes a urine metabolomic evaluation of rat model of fatty liver induced by high-fat diet and the treatment of QHD based on a GC-MS metabolomics approach combined with univariate and multivariate analyses. QHD markedly reduced liver tissue content TG apart from the lowering of serum levels of ALT, AST, and LDL. QHD significantly ameliorates the histological features of high-fat diet-treated rats. All these results suggested that intake of QHD may be useful in preventing and improving fatty liver induced by high-fat diet. Furthermore, metabolomics analysis indicated that QHD effectively regulates the perturbed metabolism by reversing changes of twelve small-molecule metabolites (especially three metabolites, tryptophan, uridine, and phenylalanine) (Table 1) and three metabolic pathways (phenylalanine, tyrosine, and tryptophan biosynthesis, phenylalanine metabolism, and tryptophan metabolism) (Figure 11).

Our findings showed that metabolic changes of QHD treatment on fatty liver are predominantly related to abnormal amino acid metabolism (phenylalanine, tyrosine, and tryptophan biosynthesis, phenylalanine metabolism, and tryptophan metabolism). In present work, the levels of amino acids (tryptophan, phenylalanine) were increased in model group compared with the normal control group. Most amino acids are synthesized and degraded in the liver; therefore, liver injury can lead to abnormal amino acid metabolism and release of amino acids from liver cells [33]. Tryptophan and phenylalanine are an aromatic amino acid, a metabolite of the intestinal flora that breaks down polyphenols and proteins in food [34]. Under the catalysis of phenylalanine deaminase, phenylalanine is converted to phenylpyruvic acid and ammonia through deamination. Phenylpyruvic acid is further converted to benzoic acid and phenylacetic acid through decarboxylation. Benzoic acid and phenylacetic acid eventually form succinic acid, which enters the tricarboxylic acid cycle [35]. Malic acid is an intermediate substance in the tricarboxylic acid cycle. In this study, the malic acid content of the model group was decreased, indicating suppressed TCA cycle, and TCA cycle was disturbed by high-fat diet. In this study, tryptophan and phenylalanine were increased in model group. The reason for the analysis may be that after the liver is damaged, the tricarboxylic acid cycle in the liver is inhibited, and the inactivation and scavenging ability of the aromatic amino acid is decreased, resulting in a significant increase in the concentration of aromatic amino acids in the urine [36].

Therefore, the level of amino acid in urine of model group was higher than that of normal control group. QHD treatment could successfully reverse the raise of the above 2 amino acids, indicating that amino acid metabolism might be connected with the therapeutic mechanism of QHD.

The alteration in levels of glycine in fatty liver rats was observed in our study. Glycine is one of the substances in the synthesis of GSH. GSH is an antioxidant molecule produced by several tissues in response to oxidative stress and increased production of reactive oxygen species (ROS) [37]. Oxidative stress is one of the mechanisms leading to liver injury, characterized by mitochondrial dysfunction, oxidative damage, and ROS production [38]. Glycine has the potential to act as a hepatospecific antioxidant to reduce oxidant and cytokine production by Kupffer cells and promote hepatic fatty acid oxidation [39]. Increasing glutathione biosynthesis by glycine supplementation to the diet of SF rats may protect the liver from oxidative stress and IR [40]. Therefore, supplemental glycine may be protective in NAFLD [39]. In the present study, glycine was significantly decreased in model group compared with the control group, which suggests that high-fat diet can cause the dysfunction of amino acid metabolism. QHD intervention of high-fat diet treated rats showed a tendency of bringing the level of glycine. Based on these findings, it is likely that QHD produced a major metabolic effects on amino acid.

Among these metabolites, butyrate and oleic acid were significantly changed in our study. Butyrate belongs to a volatile short chain fatty acid (SCFA) that is an endogenous substance of the human body, which comes from the fermentation of undigested food by the colonic microbiota. SCFA is an energy source of intestinal microorganisms and host intestinal epithelial cells, which can promote cell growth, reduce the pH value of the colon environment, and lower the growth of harmful bacteria. Supplemental SCFA can improve glucose and lipid metabolism disorders associated with obesity and may become a new therapeutic strategy for obesity-related diseases [41]. The content of oleic acid in model group was lower than that in normal control group, indicating that the synthesis of free fatty acids (FFA) increased in liver cells of rat. In the case of FFA accumulation in the liver, when lipids cannot be stored in the situation of high-fat diet or abnormal adipose tissue function, excess lipids directly enter into the liver and accumulate in the liver, causing liver inflammation and NAFLD [42]. In this paper, butyrate and oleic acid were significantly decreased in model group compared with the control group, while increase of them was shown in QHD group. These results suggested that high-fat diet affects fatty acids metabolism and QHD has a significant antihepatic fat effect by regulating abnormal fatty acid metabolism.

In this work, the decreased levels of malic acid and glucose and increased level of L-lactic acid were presented in the model group compared with the control group. The finding is mostly associated with disordered energy metabolism. Lactic acid is an end product of glycolysis, which is traditionally indicative of adverse consequence [43]. The increase of lactic acid content of urine in the model rats indicated that aerobic metabolism in vivo was inhibited due to the cause of modeling, thus promoting glycolysis, which was the manifestation of liver damage [44]. After the intervention of QHD, the dysfunction of lactate synthesis and secretion caused by modeling was obviously adjusted, and the metabolic network returned to normal range. Malic acid is the intermediate in the tricarboxylic acid cycle. The decreased level of malic acid in the model group indicates that the tricarboxylic acid cycle is inhibited and the energy metabolism in mitochondria is affected [44]. On the other hand, the inhibition of TCA cycle was alleviated by QHD, accompanied by an increase in malic acid level. Glucose was decreased in model group, which indicates the increase of energy demand and may be related to the observed alterations in the levels of metabolites participating in Kreb's cycle [45]. However, QHD intervention of high-fat diet treated rats showed a tendency of bringing the level of glucose and did not return to the level of the normal group, which was lower than that of the normal group; it is speculated that the mechanism of action QHD treatment of fatty liver is related to abnormalities in glucose metabolism

Uridine is a pyrimidine nucleoside composed of pyrimidine and ribose. The balance of uridine in the body is mainly regulated by uridine phosphorylase, and uridine can prevent fatty liver caused by some drugs. Fat metabolism in the liver can be modulated by modulating uridine phosphorylase or exogenous uridine supplementation to alter the uridine content in the body [46]. Uridine can cause insulin resistance, resulting in the failure of glucose to be used in time, resulting in high blood sugar and diabetes in severe cases. Excessive glucose enters the liver and undergoes a series of metabolisms to form triglycerides (TG), and too much TG accumulates in the liver, forming fatty liver [47]. In this study, the uridine content of urine in the model rats was significantly higher than that of the normal control group. After QHD treatment, the increased level of uridine was downregulated, suggesting that QHD may improve the pathological state of fatty liver model rats by regulating the metabolism of purine nucleotides.

Inositol is a kind of “biotin” that participates in metabolic activities in the body and has various functions such as immunization, prevention, and treatment of certain diseases [48]. Inositol plays an important role in the structural basis of eukaryotic second messengers (phosphatidylinositol, inositol phosphate, phosphoric acid, etc.) [49]. Therefore, inositol regulates intracellular calcium concentration, insulin signaling, and fatty acid oxidation [49]. The content of inositol in the model group was significantly increased, suggesting that high-fat diet feeding may cause insulin signal transduction dysfunction and fatty acid metabolism disorder in rats. After QHD treatment, the inositol content of QDH group was significantly reduced, indicating that QHD can regulate insulin signaling and fatty acid metabolism.

## 5. Conclusions

In this study, serum biochemistry, histopathology, and GC/MS-based on metabolomic analysis confirmed the antihepatic fat effect of QHD. In addition, 12 significantly disrupted biomarkers in rat urine among groups were identified as involved in phenylalanine, tyrosine, and tryptophan biosynthesis, phenylalanine metabolism, glycine, serine, and threonine metabolism, and tryptophan metabolism. These potential biomarkers and their corresponding pathways may help to further understand the mechanism of QHD in treating liver fat. Our study also showed that the established urine metabolomics method could provide a promising method for exploring the complex mechanism of Chinese herbal prescriptions.

## Figures and Tables

**Table 1:** Significantly changed metabolites.

Metabolites	Model group^a^	QHD group^b^
Butyrate	↓*∗∗*	↑
Malic acid	↓*∗∗*	↑
Glycine	↓*∗∗*	↑
L-lactic acid	↑*∗∗*	↓
Indole	↑*∗∗*	↑
Tryptophan	↑*∗*	↓#
Glucose	↓*∗*	↑
Uridine	↑*∗*	↓#
Oleic acid	↓*∗*	↑
Phenylalanine	↑*∗*	↓#
Inositol	↑*∗*	↓
Benzoic acid	↑*∗*	↓

The up or down arrows represent the relatively increased or decreased levels of the metabolites in model group or QHD group, respectively. a: compared to the control group, b: compared to the model group. *∗∗p* < 0.01, *∗p* < 0.05, # *p* < 0.05.

## Data Availability

The data used to support the findings of this study are included within the Supplementary Materials.

## Supplementary Materials

Supplementary Materials
* 2.2. Preparation of QHD. The quality control of QHD.* Our current study provides a new HPLC-based method for quality control of Qushi Huayu Decoction. The representative chemical fingerprint combined with simultaneous determination of 3 target components (polygoni, rhein, jasminoidin) offered a powerful and rational way to guarantee the quality of this herb. The results showed that extraction with water or ethanol yielded more stable components. The present study may serve as an important reference to establish the quality control method for the extracts and preparations of Qushi Huayu Decoction preparations.* 2.6. Urine Sample Preparation and Analysis. * Two hundred microliters of urine added with 30 units of urease was incubated at 37°C for 15min to decompose and remove the excess urea present in it. Then, 800*μ*l methanol and 10*μ*l myristic acid (1mgmL^−1^) used as internal standard were added to it. The solution was vigorously extracted for 1min and was centrifuged at 13,000xg, 4°C for 10min. The supernatant (200*μ*l) was transferred to a gas chromatography (GC) vial and then evaporated to dryness under nitrogen at room temperature. Methoxyamine (50*μ*l) in pyridine (15mg/ml) was added to each GC vial. The solution was then vigorously vortexed for 1min, after the methoxyation reaction at 30°C for 1.5h at room temperature. The samples were subsequently trimethylsilylated at 70°C for 1h using N-Methyl-N-(trimethylsilyl) trifluoroacetamide:trimethylchlorosilane (100:1, v/v, 50 *μ*l). Finally, 40*μ*l heptane was added to the GC vial, and the solution was vigorously vortexed again for 1min before the GC/mass spectroscopy (MS) analysis.
