# Supplementary material for: A Metabolomic Study on the Intervention of Traditional Chinese Medicine Qushi Huayu Decoction on Rat Model of Fatty Liver Induced by High-Fat Diet
Source: Biomed Res Int. 2019 Feb 7;2019:5920485. doi: 10.1155/2019/5920485 (PMC6383432; doi:10.1155/2019/5920485)
Supplement: Supplementary Materials — 2.2. Preparation of QHD. The quality control of QHD. Our current study provides a new HPLC-based method for quality control of Qushi Huayu Decoction. The representative chemical fingerprint combined with simultaneous determination of 3 target components (polygoni, rhein, jasminoidin) offered a powerful and rational way to guarantee the quality of this herb. The results showed that extraction with water or ethanol yielded more stable components. The present study may serve as an important reference to establish the quality control method for the extracts and preparations of Qushi Huayu Decoction preparations. 2.6. Urine Sample Preparation and Analysis. Two hundred microliters of urine added with 30 units of urease was incubated at 37°C for 15min to decompose and remove the excess urea present in it. Then, 800μl methanol and 10μl myristic acid (1mgmL−1) used as internal standard were added to it. The solution was vigorously extracted for 1min and was centrifuged at 13,000xg, 4°C for 10min. The supernatant (200μl) was transferred to a gas chromatography (GC) vial and then evaporated to dryness under nitrogen at room temperature. Methoxyamine (50μl) in pyridine (15mg/ml) was added to each GC vial. The solution was then vigorously vortexed for 1min, after the methoxyation reaction at 30°C for 1.5h at room temperature. The samples were subsequently trimethylsilylated at 70°C for 1h using N-Methyl-N-(trimethylsilyl) trifluoroacetamide:trimethylchlorosilane (100:1, v/v, 50 μl). Finally, 40μl heptane was added to the GC vial, and the solution was vigorously vortexed again for 1min before the GC/mass spectroscopy (MS) analysis. [file 5920485.f1.zip › 5920485/the additional material files_BMRI_2635955.docx]

**The quality control of QHD**

Chromatographic fingerprints obtained from five batches of Qushi Huayu Decoction and the reference fingerprint (mark R) developed with the median of all chromatograms are given in Fig.1and Fig.2, and the similarity values of all imported chromatograms with respect to the reference fingerprint are tabulated in Table 1. There were 14 common peaks in all 5 batches and the area sum of them accounted for above 90% of the overall peak area. Peaks 5, 8 and 13 were identified as polygoni, rhein, jasminoidin by comparing with the corresponding chemical references under the same condition (Fig.2 and Fig.3).


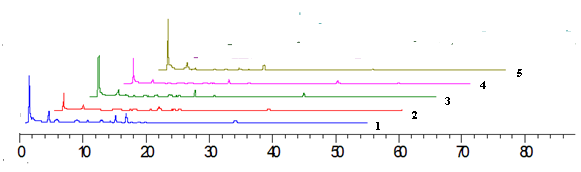


**Fig.1** Superposition of HPLC fingerprint of 5 batches of Qushi Huayu Decoction


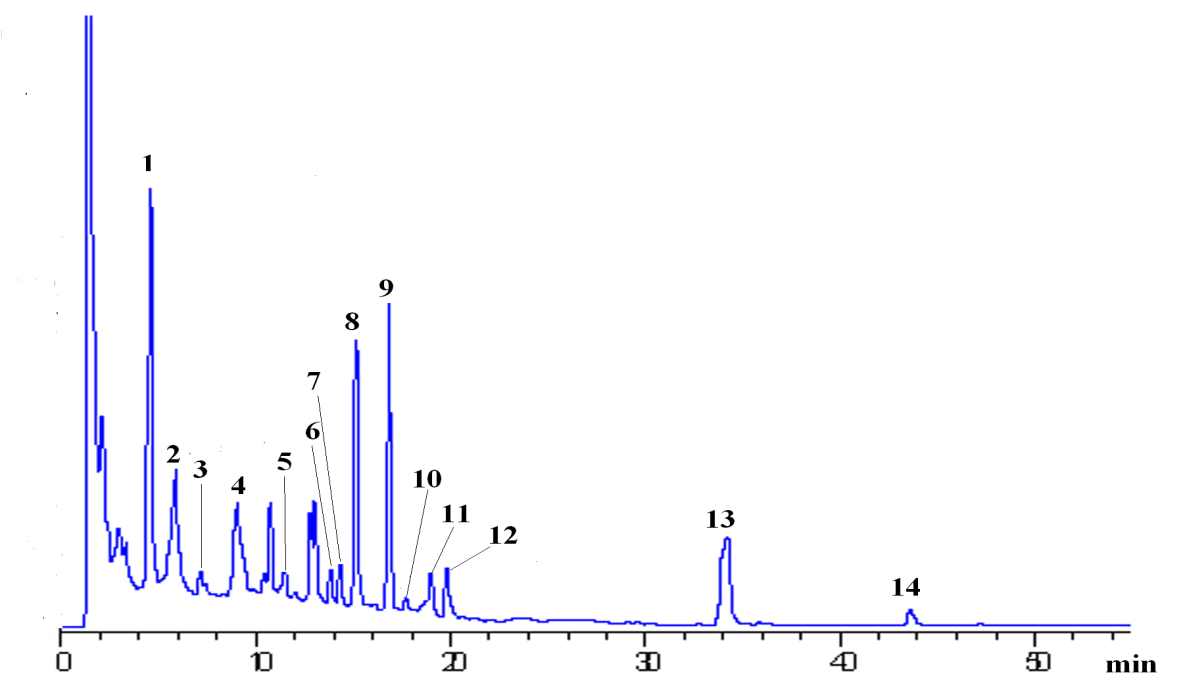


5：polygonin；8: rhein;13: jasminoidin

**Fig.2.** Chromatographic fingerprints obtained from Qushi Huayu Decoction

**
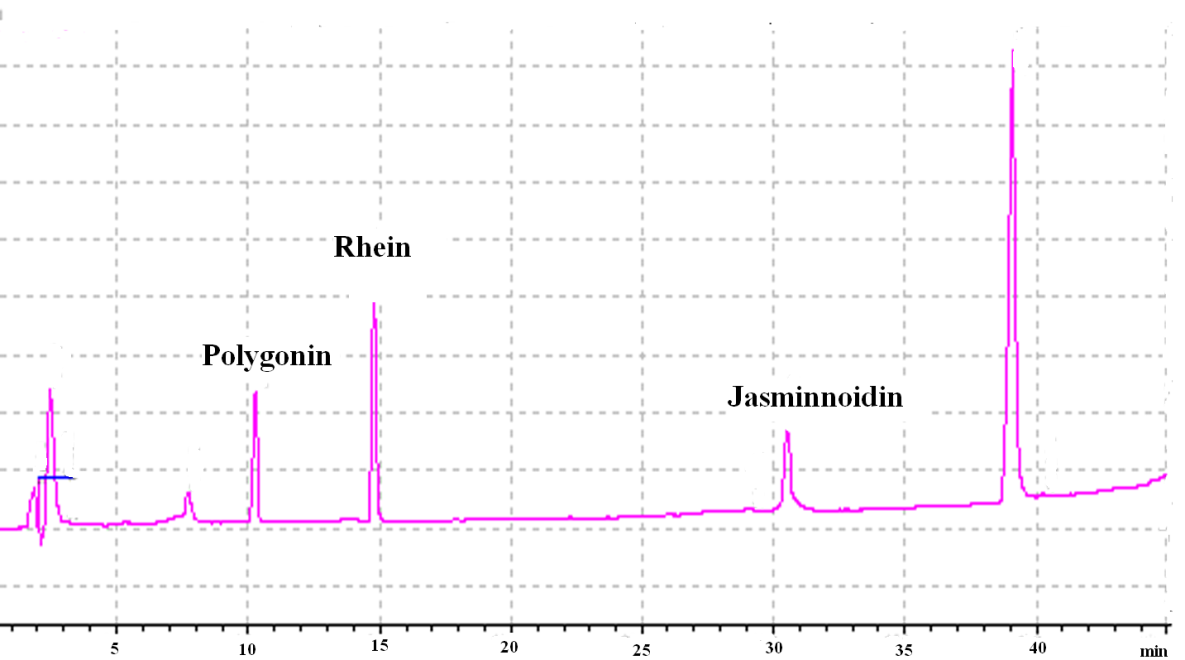
**

**Fig.3.** Chromatogram of mixed standard compounds.

**Table 1** Similar degree evaluation

| No sample Similar degree |
| --- |
| S1 0.934  S2 0.929  S3 0.95  S4 0.944  S5 0.944 |

Our current study provides a new HPLC-based method for quality control of Qushi Huayu Decoction .The representative chemical fingerprint combined with simultaneous determination of 3 target components offered a powerful and rational way to guarantee the quality of this herb. The results showed that extraction with water or ethanol yielded more stable components . The present study may serve as an important reference to establish the quality control method for the extracts and preparations of Qushi Huayu Decoction preparations.

**Urine Sample Preparation and Analysis**

Two hundred microliter of urine added with 30 units of urease was incubated at 37°C for 15min to decompose and remove the excess urea present in it. Then, 800μl methanol and 10μl myristic acid (1mgmL^-1^) used as internal standard were added to it. The solution was vigorously extracted for 1min and was centrifuged at 13,000xg, 4 ºC for 10min. The supernatant (200μl) was transferred to a gas chromatography (GC) vial, and then evaporated to dryness under nitrogen at room temperature. methoxyamine (50μl) in pyridine (15mg/ml) was added to each GC vial. The solution was then vigorously vortexed for 1min, after the methoxyation reaction at 30ºC for 1.5h at room temperature. The samples were subsequently trimethylsilylated at 70ºC for 1h using N-Methyl-N-(trimethylsilyl) trifluoroacetamide:trimethylchlorosilane (100:1, v/v, 50 μl). Finally, 40μl heptane was added to the GC vial, and the solution was vigorously vortexed again for 1min before the GC/mass spectroscopy (MS) analysis.
